# A holistic review of buffalo productivity, reproductive efficiency, genetic improvement, and disease management in Bangladesh

**DOI:** 10.1016/j.vas.2025.100496

**Published:** 2025-08-13

**Authors:** Eshtiak Ahamed Pehan, Manik Miah, Md Habibur Rahman, Shahanaj Ferdousi Shejuty, Md Nurul Haque, Md Nazmul Huda, Md Rezwanul Habib, Md Younus Ali

**Affiliations:** a Dairy Research and Training Center, Bangladesh Livestock Research Institute, Savar, Dhaka 1341, Bangladesh; b Buffalo Production Research Division, Bangladesh Livestock Research Institute, Savar, Dhaka 1341, Bangladesh; c Animal Health Research Division, Bangladesh Livestock Research Institute, Savar, Dhaka 1341, Bangladesh; d Faridpur Regional Station, Bangladesh Livestock Research Institute, Bhanga, Faridpur, Bangladesh; e Biotechnology Division, Bangladesh Livestock Research Institute, Savar, Dhaka 1341, Bangladesh; f Goat Production Research Division, Bangladesh Livestock Research Institute, Savar, Dhaka 1341, Bangladesh; g Department of Dairy Science, Bangladesh Agricultural University, Mymensingh, 2202, Bangladesh; h Department of Animal Breeding and Genetics, Bangladesh Agricultural University, Mymensingh, 2202, Bangladesh

**Keywords:** Buffalo, Breeding strategies, Genetic improvement, Reproductive efficiency, Health management, Bangladesh

## Abstract

Buffaloes play a vital role in Bangladesh's livestock sector, contributing significantly to the nation’s milk and meat production. However, their productivity remains below potential due to limited genetic capacity, poor reproductive performance, and inadequate health and management practices. This review critically synthesizes findings from scientific literature, field studies, and national reports to assess the status of buffalo production, reproductive efficiency, genetic improvement efforts, and disease management strategies in Bangladesh. Major challenges include low milk yield (average 2.50-4.00 liters/day), imbalanced nutrition, reliance on traditional feeding systems, and minimal mechanization. Reproductive inefficiencies are characterized by low conception rates (below 40%), prolonged calving intervals (local:19.36 ± 2.39 months; crossbred: 19.37 ± 2.63 months), delayed onset of puberty (30 to 36 months), and ineffective estrus detection. Although crossbreeding programs with high-yielding breeds such as Murrah and Nili-Ravi have been introduced, progress has been limited due to inadequate record-keeping, lack of performance monitoring, and continued dependence on conventional breeding methods. Disease prevention and control are further impeded by insufficient veterinary infrastructure, low vaccination coverage, and limited farmer awareness. Structural barriers such as the absence of integrated development frameworks, restricted access to artificial insemination (AI), and a shortage of superior germplasm also hinder sectoral advancement. To address these multifaceted issues, the review advocates for enhanced farmer education, expansion of AI services, development of region-specific disease control strategies, and the implementation of systematic genetic improvement programs incorporating molecular technologies. Strengthened collaboration among government agencies, research institutions, and farming communities is essential to foster a resilient, productive, and sustainable buffalo industry in Bangladesh.

## 1 Introduction

**Fig. 1:**
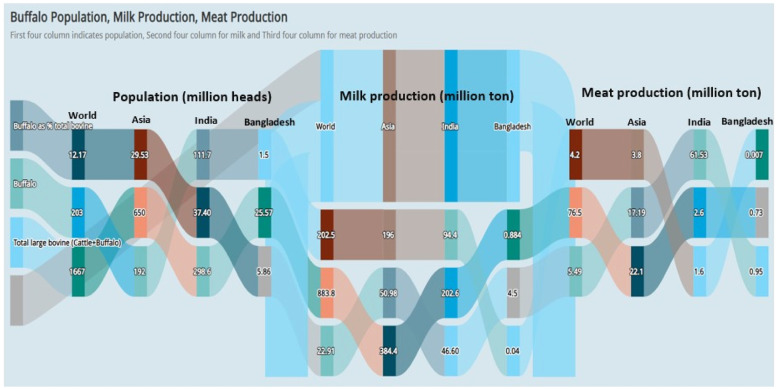
Sankey diagram showing the global distribution of buffalo population, milk, and meat production, highlighting Asia, especially India, dominates globally, while Bangladesh contributes modestly (FAO, 2021 and other regional statistics).

Buffalo *(Bubalus bubalis)* is an important domesticated livestock species in Bangladesh, playing a significant role in rural livelihoods and contributing to the national economy (Habib et al., 2017). The current buffalo population is approximately 1.508 million, predominantly of indigenous origin (DLS, 2024). Two subspecies are found in the country: The Riverine type (*Bubalus bubalis bubalis*) and the Swamp type (*Bubalus bubalis carabanensis*), which differ in morphology and chromosome number. Riverine buffaloes possess 50 chromosomes, while Swamp buffaloes have 48 (Hamid et al., 2017). Among the Riverine breeds, Murrah, Nili-Ravi, Surti, and Jaffarabadi are famous for their superior milk production. Crossbreeding between indigenous and neighboring Indian breeds occurs along the border areas due to transboundary animal movement (Hamid et al., 2016a). Approximately 40.00% of the buffalo population resides in coastal regions, with the rest distributed across the Meghna-Ganga and Jamuna-Brahmaputra floodplains. Buffaloes in Bangladesh are raised under various management systems: extensive (56.00%), semi-intensive (42.00%), and intensive (2.00%) (Sultana et al., 2024). Despite their adaptability and production potential, buffaloes contribute only 1.40% to the national milk output and 0.95% to meat production, far below the levels observed in neighboring countries such as India (51.20%), Pakistan (59.50%), Nepal (66.60%), and Sri Lanka (18.00%) (Hamid et al., 2016b; Siddiky & Faruque, 2017). According to global estimates, buffaloes contribute significantly to milk and meat production, particularly in India, while Bangladesh lags behind (Fig. 1).

Additionally, buffalo is the second most important livestock species in Bangladesh after cattle, with significant contributions to rural livelihoods and the national economy (Hamid et al., 2016b). Their ability to utilize poor-quality roughage, withstand harsh environmental conditions, and yield relatively high economic returns makes them ideal for sustainable livestock development. However, they have historically been neglected in terms of policy, research, and development support. In contrast, buffaloes have played a central role in the dairy industry in countries like India. According to FAO (2021), global trends in buffalo production are on the rise, particularly in Asia and South Asia, including Bangladesh. Several challenges hinder buffalo productivity in Bangladesh. These include inadequate feed and fodder availability, declining grazing land, insufficient access to clean water and veterinary services, low genetic merit, and limited application of reproductive technologies such as artificial insemination (AI) (Islam et al., 2017; Pehan et al., 2022). Addressing these issues necessitates a thorough understanding of both genetic and non-genetic factors that influence key economic traits. Partitioning phenotypic variation into genetic and environmental components is essential for effective breeding program design (Abou-Bakr, 2009). Performance traits like milk yield and fertility are also influenced by environmental factors such as season of calving and parity (Aziz et al., 2001; El-Arian et al., 2012; Hassan et al., 2017). Given that reproductive efficiency and milk production are major determinants of dairy profitability, improving these parameters is imperative (LeBlanc, 2010). Conserving and improving indigenous buffalo germplasm is vital for long-term productivity enhancement (Aminafshar et al., 2008). Crossbreeding with high-performing exotic breeds holds great potential, but breeding strategies need to be adapted to the country’s diverse agro-ecological zones. Assisted reproductive technologies (ARTs) like embryo transfer, multiple ovulation and embryo transfer (MOET), ovum pick-up (OPU), and in vitro fertilization (IVF), estrous synchronization, AI, can accelerate genetic improvement (Kumar et al., 2024; Muner et al., 2025). However, adoption remains limited due to seasonal breeding tendencies and low conception rates. Furthermore, buffaloes, especially the Riverine type, are known for their adaptability to varied topographies and climates but remain vulnerable to several diseases and health disorders that can negatively impact production and lead to mortality (Villanueva et al., 2018). Therefore, any genetic improvement strategy must be integrated with robust health management systems. Effective health interventions, including vaccination, regular health screenings, biosecurity measures, and parasite control, are crucial to improving reproductive performance and reducing economic losses (Jadav et al., 2022; Dodiya et al., 2024). Proper health management not only ensures feed efficiency and production sustainability but also prolongs the productive lifespan of breeding stock.

Although buffaloes are increasingly important in Bangladesh's livestock sector, no comprehensive review currently synthesizes the interconnected domains of productivity, reproduction, genetics, and health management. Existing literature often addresses these areas in isolation and lacks contextual integration. Considering rising demand for milk and meat, environmental constraints, and the growing accessibility of advanced technologies, a holistic and evidence-based evaluation is urgently required. Therefore, this review aims to provide a comprehensive assessment of buffalo productivity, reproductive efficiency, genetic improvement, and disease management in Bangladesh. It identifies current limitations, explores technological advancements, and offers strategic recommendations for developing a sustainable and health-resilient buffalo production system suited to the specific context of Bangladesh. Additionally, this manuscript takes a structured narrative approach, combining research findings, national statistics, and expert perspectives to provide a holistic and integrated view of advanced buffalo production, reproduction, genetics, and buffalo health management in Bangladesh.

## 2 Methodology

### 2.1 Data collection

Though not a systematic review, this narrative review used a structured and transparent literature search and inclusion process to ensure replicability. A systematic approach was adopted to collect relevant literature on buffalo genetics, breeding strategies, and health management practices in Bangladesh. Sources included peer-reviewed scientific articles, government reports, and publications from recognized national and international organizations, such as the Department of Livestock Services (DLS), the Food and Agriculture Organization of the United Nations (FAO), and the International Buffalo Federation. To ensure the inclusion of up-to-date and high-quality literature, extensive literature searches were conducted using electronic databases, including PubMed, Scopus, Web of Science, and Google Scholar. Search terms were applied using specific keywords and Boolean operators, such as: “Buffalo breeding in Bangladesh,” “Genetic improvement in buffalo,” “Crossbreeding with riverine breeds,” “Reproductive technologies in buffalo,” “Buffalo milk and meat production,” “Prevalent diseases in buffalo,” “Buffalo Health Management,” and “Buffalo Health and Productivity”. The inclusion criteria focused on publications from 2000 to 2025, with emphasis on studies relevant to the agro-ecological conditions and socio-economic context of Bangladesh.

### 2.2 Literature review

A structured review was conducted to assess current literature on buffalo breeding, genetic improvement, and health management in Bangladesh. The analysis incorporated peer-reviewed journal articles, national reports, and case studies, focusing on core areas such as crossbreeding strategies, genetic evaluation techniques, reproductive biotechnology, and enhancements in milk and meat production.

Emphasis was placed on evaluating studies involving crossbreeding with high-yielding Riverine breeds (e.g., Murrah, Nili-Ravi) to determine their impact on the genetic performance and productivity of indigenous buffalo populations. Research on reproductive biotechnologies-including artificial insemination (AI), estrus synchronization (ES), and in vitro fertilization (IVF) was reviewed to assess their adaptability and effectiveness under local production systems.

Health management practices were analyzed for their influence on reproductive efficiency, feed conversion, disease control, and herd productivity. Particular attention was given to the relationship between infectious disease prevalence, mortality rates, and the implementation of preventive veterinary interventions. The integration of genetic improvement programs with comprehensive health management was recognized as a critical component of sustainable buffalo production systems. This review identifies recent innovations, persistent constraints, and emerging trends relevant to improving buffalo productivity, particularly in terms of milk and meat yield, providing evidence-based insights for strategic development in the Bangladeshi context.

### 2.3 Inclusion and exclusion criteria

**Fig. 2:**
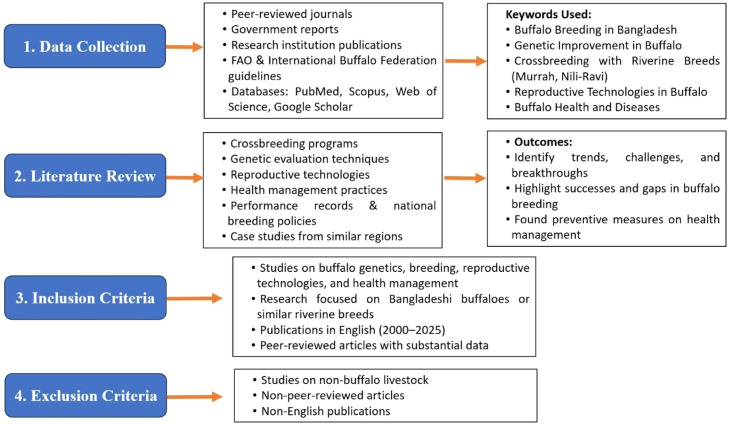
Flow diagram illustrating the systematic process used for data collection, literature screening, and application of inclusion and exclusion criteria in the preparation of this review.

To maintain the relevance, scientific rigor, and focus of this review, clearly defined inclusion and exclusion criteria were applied during the selection of literature. Studies were included if they addressed key aspects of buffalo genetics, breeding strategies, and reproductive technologies, or with particular emphasis on research involving Bangladeshi buffalo populations or closely related riverine breeds like Murrah and Nili-Ravi. Priority was given to peer-reviewed publications in English, published between 2000 and 2025, to ensure the incorporation of current and credible information. Conversely, studies were if they focused on livestock species other than buffalo, lacked peer review or a clearly defined methodology, did not present substantial or relevant data, were published in languages other than English, or were identified as duplicate records across different databases (Fig. 2).

## 3 Buffalo genetic resources in Bangladesh and their population

**Fig. 3:**
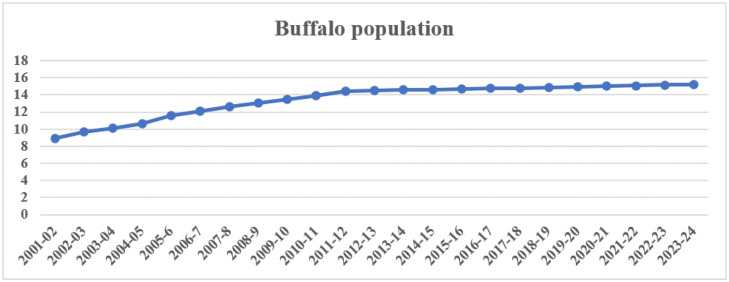
Buffalo population growth trend in Bangladesh from 2001 to 2024 (Source: DLS, 2024).

**Fig. 4:**
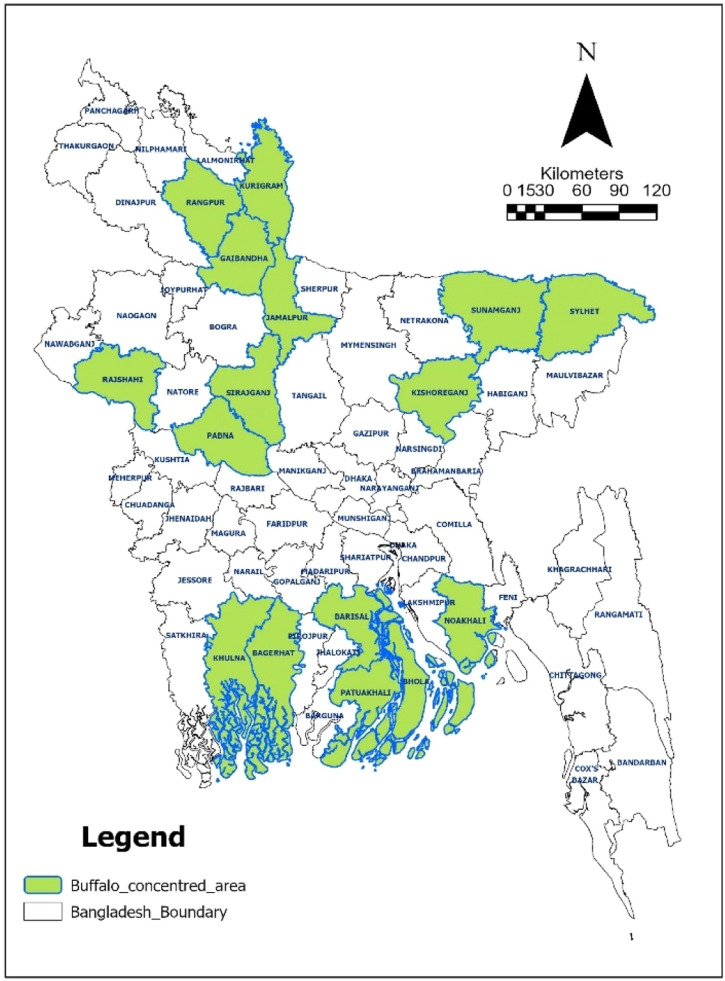
Map showing buffalo-concentrated areas in Bangladesh is highlighted in green, with a scale bar and north arrow for orientation.

**Fig. 5:**
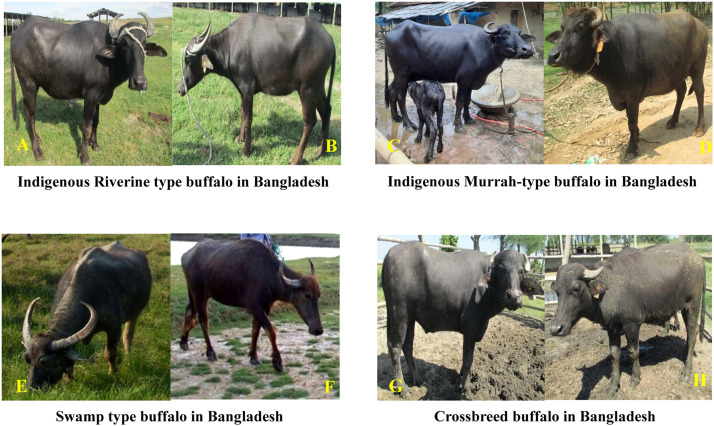
Representative images of buffalo genotypes found in Bangladesh. (A, B) Indigenous riverine-type buffaloes (C, D), indigenous Murrah-type buffaloes (E, F), swamp-type buffaloes, and (G, H) crossbred buffaloes (Hamid et al., 2017).

Bangladesh harbors several indigenous buffalo populations that play a vital role in milk production, meat supply, and draught power. The buffalo population in Bangladesh has shown a gradual increase in the last two decades (Fig. 3). These buffaloes are primarily classified into two types: riverine and swamp. Riverine buffaloes are mostly distributed in the southern coastal regions, including Khulna, Barisal, Bhola, and Patuakhali, while swamp buffaloes are predominant in hilly and floodplain areas, such as Sylhet and Sunamganj in the northeast, and in the northern river basin districts like Sirajganj, Jamalpur, and Kurigram (Fig. 4) (Hamid et al., 2016b; Sarker et al., 2024). In addition to indigenous types, purebred buffaloes such as Murrah and Nili-Ravi have been introduced in border regions through transboundary movement from neighboring India (Habib et al., 2023). Although indigenous buffaloes generally produce lower milk yields than exotic breeds, they are known for high milk fat content (6–8%) and are highly valued for their adaptability, disease resistance, and performance under semi-intensive production systems (Habib et al., 2017). An overview of the key characteristics of buffalo genotypes in Bangladesh is summarized in Table 1, while representative images of these genotypes are illustrated in Fig. 5.

**Table 1:** Overview of buffalo genotypes in Bangladesh (Rashid et al., 2019).

Type / Breed	Region	Genotypic and Phenotypic Characteristics
Indigenous (River type)	Central & Western parts	Medium-sized; jet black to black coat; 50 chromosomes
Indigenous (Swamp type)	Eastern part	Small-sized; grey coat with chevron and white markings; crescent-shaped horns; 48 chromosomes
Bangladeshi (Local Mixed Type)	Southwest & Central	Medium-sized; light black coat with chevron and white stockings; 50 chromosomes
Indigenous (River), Murrah × Indigenous Crossbred, Nili-Ravi × Indigenous Crossbred	Bangladesh Livestock Research Institute (BLRI)	Medium-sized; jet black to black coat; medium to small horns (either back coiled or medium long); brownish-black variation with white markings on forehead and limbs; 50 chromosomes
Crossbred (Murrah, Nili-Ravi types)	Southern parts, Indian border regions	Medium-sized; phenotypic traits vary depending on parent breeds (e.g., coat color, horn shape, body size); 50 chromosomes
Nili-Ravi, Murrah, Indigenous (crossbred)	Bagerhat (Buffalo Breeding Farm)	Nili-Ravi characteristics: large body 0’osize, curved horns, white markings, high milk yield potential; 50 chromosomes

## 4 Production performance of buffaloes in Bangladesh

Buffalo farming is an important component of Bangladesh’s livestock sector, particularly in rural floodplains, haor ecosystems, and coastal zones, where buffaloes exhibit strong adaptability to environmental stressors (Samad, 2020). While indigenous buffalo genotypes are still predominant, there is a growing trend toward crossbreeding with genetically superior breeds such as Murrah and Nili-Ravi to improve productive performance traits, especially milk and meat yield (Gowane & Vohra, 2022). However, challenges such as endemic diseases, feed shortages, and genetic limitations hinder optimal productivity (Habib et al., 2023).

### 4.1 Milk production performance

In Bangladesh, buffaloes are a valuable source of high-fat milk, containing 6.50–8.00% fat, significantly higher than the 3.50–4.00% found in cow milk (Habib et al., 2017). The average daily milk yield of indigenous buffalo ranges from 2.50 to 4.00 liters but can be increased to 6.00 to 10.00 liters through crossbreeding with superior breeds like Murrah and Nili-Ravi (Hamid et al., 2016b; Islam et al., 2017; Samad, 2020). Lactation length in indigenous buffalo spans 270–300 days, whereas crossbred buffaloes exhibit extended lactation periods of 300–330 days (Kabir et al., 2017; Omar et al., 2024). Prominent buffalo milk-producing areas include Noakhali, Bhola, and Patuakhali (Habib et al., 2023). A recent study recorded an average daily milk yield of 3.00 liters and a lactation period of 179.90 ± 3.84 days (Habib et al., 2023). Despite genetic and management improvements, milk production remains suboptimal due to nutritional deficiencies, limited veterinary services, and insufficient adoption of modern husbandry practices (Hamid et al., 2016a).

### 4.2 Meat production performance

Buffalo meat, known locally as buff, is becoming increasingly popular in Bangladesh due to its leanness (fat 1.15%) and lower cholesterol (46 mg/100 g) compared to beef (fat 4.33%; cholesterol 70 mg/100 gm) (Naveena et al., 2014). Indigenous buffaloes generally reach a mature live weight of 300–400 kg by 3–4 years of age, while crossbred buffaloes, including Murrah and Nili-Ravi types, can attain 500–600 kg under improved feeding and management systems (Islam et al., 2017; Saha et al., 2018). The carcass dressing percentage typically ranges from 50% to 55%, reflecting their potential for meat production (Maheswarappa et al., 2022). However, the slow growth rate (250–350 g/day) of indigenous genotypes remains a significant constraint to enhancing buffalo meat productivity (Karim et al., 2023).

## 5 Reproductive efficiency of buffaloes in Bangladesh

Buffaloes are valuable livestock in Bangladesh, yet their reproductive performance remains markedly lower than that of cattle. Key limitations include delayed onset of puberty (Buffaloes is around 36-42 months compared to 18-24 months in cattle), prolonged calving intervals (buffaloes 18-24 months versus cattle 12-14 months), low conception rates (buffaloes are typically 30–45% compared to 50–60% in cattle), postpartum anestrus, and distinct seasonal breeding patterns (Rahman et al., 2019; Mohammed et al., 2018). These reproductive inefficiencies are attributed to a combination of genetic, nutritional, environmental, and managerial factors.

### 5.1 Puberty and sexual maturation

The onset of puberty in buffalo heifers in Bangladesh is considerably delayed, typically occurring between 30 to 36 months (Jahan et al., 2024), which is later than buffaloes managed under improved farming systems (18 to 24 months). This delay is primarily attributed to poor nutrition, environmental stress, and inadequate management (Rahman et al., 2019; Rahman et al., 2020). Puberty is influenced by several factors, including genotype, climate, nutrition, and husbandry practices. In comparison, under improved conditions, breeds such as Murrah and Nili-Ravi reach puberty earlier, typically between 24 and 30 months (Perera, 2011). According to Jainudeen & Hafez (2000), buffaloes typically attain puberty at 60% of their adult body weight (250 - 400 kg), with age at puberty ranging from 18 to 46 months.

### 5.2 Silent estrus, estrous behavior, and detection

Buffaloes often display weak or silent estrus signs, making visual detection difficult and contributing to missed breeding opportunities and longer calving intervals (Ahmed et al., 2012a). Estrus in buffaloes typically lasts 12–24 h and is more visible during cooler months (Jainudeen & Hafez, 2000; Barile, 2005), contributing to seasonal breeding patterns. The hot and humid climate of Bangladesh further reduces the visibility of estrous signs, complicating timely detection by farmers (Sarder et al., 2007). Estrus synchronization using hormonal treatments such as prostaglandins and GnRH has shown effectiveness (Akhter et al., 2013), yet adoption remains limited due to high costs and inadequate technical knowledge among rural farmers (Rashid et al., 2019).

### 5.3 Age at first calving

The average age at first calving for indigenous buffaloes under traditional management is 42–48 months (Rashid et al., 2019), while crossbred buffaloes may calve slightly earlier, around 36–40 months (Rahman et al., 2020). Additionally, Rashid et al. (2019) reported first calving ages for local, crossbred, Nilli, and Murrah buffaloes in Bangladesh as 46.12 ± 1.66, 46.56 ± 1.64, 46.18 ± 0.88, and 46.25 ± 1.81 months, respectively. Delayed attainment of puberty and first calving significantly reduces the reproductive lifespan and productivity of female buffaloes.

### 5.4 Postpartum anestrus

Postpartum anestrus, defined as the absence of estrous cycles following parturition, presents a major barrier to reproductive efficiency in buffaloes. This anestrous interval often exceeds 120 days and is commonly associated with poor body condition, hormonal imbalance, and physiological stress (Barile, 2005; Qureshi et al., 2007). Paul and Prakash (2005) reported postpartum estrus intervals of approximately 153 days in buffaloes from Pirojpur and Barguna districts. Similarly, El-Wishy (2007) recorded an average interval of 146.2 days, while Rashid et al. (2019) observed postpartum estrus resumption ranging from 30 to 171 days across different genotypes. Habib et al. (2023) recorded the postpartum heat period as 90 days in the Lalmonirhat district. Hormonal interventions, including GnRH and progesterone-based protocols, have shown potential in reducing the anestrous period (Haque et al., 2020); however, limited accessibility and infrastructure in rural regions constrain their widespread application.

### 5.5 Calving interval

In traditional buffalo production systems in Bangladesh, calving intervals commonly range from 16 to 18 months, which exceeds the optimal 12 to 14 months necessary for maximum reproductive efficiency (Islam et al., 2017; Rashid et al., 2019). Extended calving intervals reduce both the number of calves produced and the total milk yield over the productive lifespan of the animal. Rashid et al. (2019) reported mean calving intervals of 19.36 ± 2.39 months in local buffaloes, 19.37 ± 2.63 months in crossbred buffaloes, 19.41 ± 1.66 months in Nilli Ravi, and 18.31 ± 1.97 months in Murrah buffaloes. Similarly, Habib et al. (2023) recorded an average calving interval of 478 days in buffaloes from northern Bangladesh.

### 5.6 Conception rates and pregnancy maintenance

**Fig. 6:**
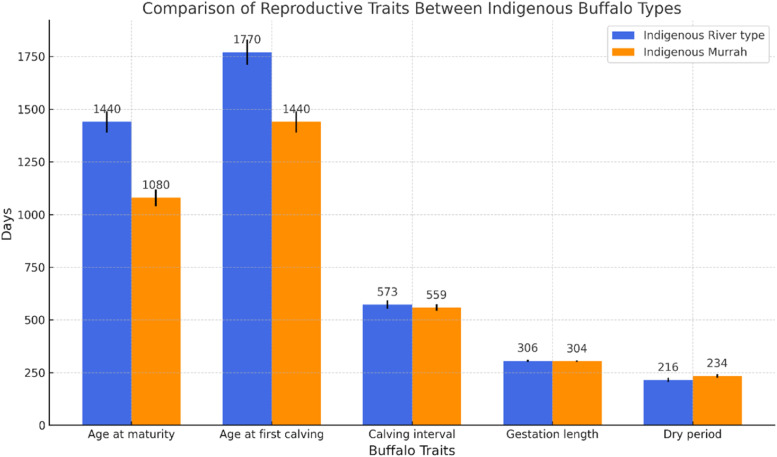
Average values on reproductive performances of buffaloes in Bangladesh.

Conception rates in buffaloes vary from 45% to 55% under natural mating but decline to 30% to 40% when artificial insemination (AI) is employed (Mohammed et al., 2018). Low AI success is attributed to poor estrus detection, improper timing, poor semen quality, and inadequate insemination techniques. Additionally, embryonic mortality is relatively high during early gestation, ranging from 15% to 20%, often resulting from nutritional deficiencies, infectious diseases, and endocrine imbalances (Khan et al., 2009). To improve reproductive outcomes, it is critical to enhance nutritional management, reproductive health protocols, and access to veterinary reproductive services. A summary of reproductive performance indicators is illustrated in Fig. 6.

### 5.7 Fertility management and nutritional factors

Nutrition is a key determinant of reproductive function in buffaloes, with ovarian inactivity commonly resulting from nutritional deficiencies (Qureshi, 2007; Vale et al., 2019). In Bangladesh, buffaloes predominantly graze on low-quality communal pastures, particularly in bathan systems, which limits their nutrient intake (Uddin et al., 2016). Nutritional supplementation during the transition period has been shown to enhance reproductive efficiency; Habib et al. (2020) reported that concentrate feeding reduced postpartum anestrus (90 days) duration and open days (90 to 120 days). Singh et al. (2022) demonstrated that molasses supplementation in Murrah calves accelerated puberty onset (24–30 months) and improved semen quality (volume: 2.35±0.10 ml; concentration: 985.30±52.42; individual motility: 65.67±2.01). While Afroz et al. (2020) observed no significant effect of varying concentrate levels on age at puberty, other studies highlight the reproductive benefits of supplementing diets with α-tocopherol, vitamin E, selenium, niacin, and bypass fats (Sarwar et al., 2009; Ganie et al., 2014).

### 5.8 Seasonality and environmental factors

The tropical climate of Bangladesh, characterized by temperatures between 32 and 40°C and high humidity, imposes significant heat stress on buffaloes, suppressing secretion of luteinizing hormone (LH), follicle-stimulating hormone (FSH), progesterone, and estradiol (Table 2). This hormonal disruption adversely affects estrus expression and embryonic development (Alam et al., 2010; Habib et al., 2017; Krishnan et al., 2017). Buffaloes exhibit greater reproductive activity during cooler seasons, whereas prolonged heat reduces estrus frequency and conception rates (Chawicha & Mummed, 2022; Omar et al., 2024). Cooling interventions such as showering buffalo bulls have been shown to improve semen quality by enhancing sperm motility and volume (Hoque et al., 2011). Management practices such as cooling systems, fixed-time AI, additional water, and nutritional supplementation during hot periods can mitigate the negative impacts of heat stress (Balhara et al., 2017; Fournel & Charbonneau, 2017).

**Table 2:** Impact of heat stress on reproductive performance in buffaloes.

Physiological Aspect	Effect of Heat Stress	Reproductive Consequences	Researchers
Feed intake	Reduced voluntary feed intake up to 10-30% when THI >75	Sperm production declined by 13.16% (from 3.95 ± 0.33 ml in neutral conditions to 3.43 ± 0.21 ml under heat stress), with prolonged reaction time and disrupted cycles.	Roth (2017); Bhakat et al. (2009)
Endocrine function	Induce hormonal disruptions	Conception rate decreased by 15-25%**,** calving interval extended by 30-50 days	Khan et al. (2023)
Gonadotropin secretion	Suppressed release of FSH and LH	Impaired spermatogenesis and ovarian inactivity	Habib et al. (2017)
Estradiol synthesis	Inhibited estradiol production	Reduced intensity and frequency of estrus expression	Pasha et al. (2024)
Prolactin concentration	Elevated circulating prolactin levels	Irregular estrous cycles and impaired reproductive rhythms	Dash et al. (2016)
Core body temperature	Increased due to environmental heat load	Cellular dysfunction in the female reproductive tract	Ahmad et al. (2020)

Given the detrimental effects of heat stress on reproductive physiology in buffaloes, various nutritional interventions have been explored to mitigate these impacts. These strategies aim to enhance thermotolerance, support hormonal regulation, and improve reproductive outcomes. Given the significant impact of heat stress on the reproductive efficiency of buffaloes, several feed supplementation strategies have been proposed to alleviate its negative effects. These dietary interventions aim to improve the animal's physiological resilience to heat, support endocrine balance, and enhance reproductive functions. Vitamins, minerals, antioxidants, and natural additives have shown promising results in minimizing the impact of high temperatures on reproductive parameters such as hormone levels, estrus expression, and conception rates. Table 3 summarizes the commonly recommended feed supplements for reducing heat stress and improving the reproductive performance of buffaloes.

**Table 3:** Nutritional interventions to mitigate heat stress in buffaloes.

Feed Additive	Physiological Function	Investigators
Vitamins A, C, E; Zn, Cu, Se, Na, NaHCO₃	Enhance antioxidant defense and maintain electrolyte homeostasis	Dash et al. (2016)
Bypass fat	Improves energy balance and supports reproductive efficiency	Ahmad et al. (2020)
Moringa oleifera leaves	Provide phytogenic antioxidants and modulate endocrine responses	Wafa et al. (2017)
Vitamins E, A, and Selenium	Reduce oxidative stress and support reproductive function	Das (2018)
Beta-carotene	Stimulates ovarian activity and enhances conception rates	Mitsuishi & Yayota (2024)
Sodium bicarbonate	Buffers ruminal pH and promotes dry matter intake	Sarwar et al. (2007)
Melatonin	Regulates circadian rhythms and reproductive hormone secretion	Domple et al. (2021)
Yeast, Niacin, Zn, and Chromium	Enhance metabolic adaptation and immune function under heat stress	Purwar & Dang (2017)

## 6 Breeding and selection of buffaloes in Bangladesh

Selective breeding of dairy buffaloes is fundamental for increasing milk yield and enhancing the livelihoods of rural communities in Bangladesh. Despite their significant economic and cultural values, buffaloes have historically received limited attention due to the absence of systematic breeding programs and genetic improvement initiatives. This has resulted in lower productivity compared to neighboring countries such as India and Pakistan (Uddin et al., 2016; Hamid et al., 2017). To enhance buffalo production, a breeding program focused on milk yield, adaptability, body conformation, and reproductive traits, including age at first calving and calving intervals, is essential (Rashid et al., 2019). Indigenous buffaloes are relatively low milk producers, but their productivity can be enhanced through selective breeding with exotic breeds such as Murrah and Nili-Ravi, which are globally recognized for their high milk production and strong pedigree records (Dhanda, 2013; Hamid et al., 2016a; Samad, 2020). A well-structured breeding program integrating indigenous conservation with selective crossbreeding, AI and performance recording can significantly improve indigenous buffalo productivity.

### 6.1 Crossbreeding for genetic improvement

The DLS of Bangladesh initiated a crossbreeding program for indigenous buffaloes by introducing Murrah and Nili-Ravi bulls from Pakistan in the mid-19th century. Subsequently, the Buffalo Breeding Farm (BBF) in Bagerhat was established to support this effort (Hamid et al., 2017). While indigenous buffaloes are highly adaptable, they produce less milk and have shorter lactation periods compared to exotic breeds. To combine local adaptability with enhanced productivity, crossbreeding with Murrah and Nili-Ravi is considered effective due to their higher milk yields and longer lactation lengths (Rashid et al., 2019; Samad, 2020). This approach has been a key strategy in improving the productive and reproductive performance of indigenous buffaloes (Omar et al., 2024). Studies indicate that crossbred buffaloes outperform local breeds in milk production and reproductive traits under improved management (Rashid et al., 2019; Shahjahan, 2021). Since 2011, Lal Teer (LTL), a private organization, has also introduced Italian Mediterranean bull semen in its research and development farm to further support genetic improvement (Hamid et al., 2017). Thus, upgrading indigenous buffaloes using elite local females and semen from proven imported bulls presents a promising pathway for breed development in Bangladesh (Habib et al., 2017). However, appropriate planning and monitoring are vital to preserve genetic diversity and prevent increased disease susceptibility as native traits risk erosion (Hamid et al., 2017).

### 6.2 Artificial insemination (AI) in buffaloes

AI is an important tool for genetic improvement in livestock, but its use in buffalo production in Bangladesh remains very limited. Most buffaloes are still bred naturally, and AI is practiced only in selected coastal and central areas (Uddin et al., 2016; Habib et al., 2017). Several factors contribute to this low adoption, such as difficulties in detecting estrus, the seasonal breeding pattern of buffaloes, and a shortage of trained AI technicians (Hamid, 2018).

Buffaloes often exhibit silent or weak estrus signs, making it difficult for farmers to identify the optimal time for insemination. The commonly used "am-pm rule" from cattle does not always apply to buffaloes because of their different reproductive physiology (Sarker et al., 2024). Research suggests that in intensive farming conditions, insemination should preferably occur 12-18 h after the onset of estrus to achieve better conception rates (Hamid, 2018). However, in extensive or semi-intensive systems where estrus detection is often delayed, then inseminating 25-36 h after the first signs of estrus may yield better results (Rahman et al., 2020). Another major challenge is the availability and storage of semen. Although Murrah and Nili-Ravi semen are imported for crossbreeding, the scale of AI services is restricted because of inadequate semen production, limited semen bank facilities, and poor cold chain management (Rashid et al., 2019). The use of high-quality semen from improved riverine Murrah and Nili-Ravi bulls can further enhance the milk and meat production of indigenous buffaloes. A big problem is the lack of buffalo-specific AI protocols and limited extension services to support farmers (Rashid et al., 2019). To promote wider use of AI, Bangladesh needs to improve farmer training in estrus detection, enhance the semen supply chain, and develop breeding management programs specifically designed for buffalo.

### 6.3 Assisted reproductive technologies (ARTs) in Bangladeshi buffaloes

In Bangladesh, the application of ARTs in buffalo production is still at a very early stage, constraining opportunities to enhance reproductive efficiency and productivity. Conception rates often remain below 40%, primarily due to inadequate estrus detection, seasonal anestrus, and insufficient technical expertise (Hamid, 2018; Rashid et al., 2019). Hormonal protocols like Ovsynch and CIDR-based synchronization have shown good potential to improve conception rates, but their use at the field level is still very limited (Habib et al., 2023). Advanced biotechnologies such as embryo transfer, MOET, OPU, and IVF are commonly practiced in many South Asian countries like India and Pakistan (Srirattana et al., 2022; Kumar et al., 2024; Muner et al., 2025), but these technologies are almost non-existent in Bangladesh. The reasons include inadequate infrastructure, lack of skilled manpower, and limited institutional capacity. Although crossbreeding with Murrah and Nili-Ravi buffaloes has started to improve milk production, the progress is slow because of poor genetic recording and evaluation systems (Charlini & Sinniah, 2015; Habib et al., 2023). However, recent genomic studies on buffalo diversity in the region provide a solid base for future precision breeding programs (Rehman et al., 2021; Ahlawat et al., 2024). To overcome these limitations, a well-defined coordinated national approach is needed that integrates, estrus synchronization, embryo transfer, OPU-IVF, MOET, and genomics, combined with proper training (Kumar et al., 2024). This would help improve reproductive efficiency and ensure sustainable development of the buffalo sector.

### 6.4 Selection and genetic evaluation

The rich genetic diversity of buffaloes in Bangladesh is under threat due to disorganized and uncontrolled breeding practices (Hamid et al., 2017). To improve productivity, proper selection and genetic evaluation, focusing on productive, reproductive, adaptive, and disease-resistant traits, are essential. Phenotypic traits with economic significance should be prioritized, and the genetic uniqueness of indigenous buffaloes should be preserved. In a notable effort, Lal Teer Livestock Limited (LTL), in collaboration with the Beijing Genomics Institute (BGI), successfully sequenced the buffalo genome in 2011, offering new prospects for enhancing the productivity of indigenous buffaloes (Hamid et al., 2017). Despite relatively low average milk production (600–1000 L per lactation, 250–270 days), structured selection and breeding strategies hold promise for significant improvement (Habib et al., 2023). Moreover, ongoing initiatives aimed at buffalo development further underscore the commitment to boosting the productivity and sustainability of the indigenous buffalo population (Omar et al., 2024).

## 7 Major diseases of buffalo and their management

Health management is an essential component of buffalo husbandry, as disease constraints significantly reduce productivity and profitability (Jadav et al., 2022). The hot and humid climate of Bangladesh further predisposes a variety of infectious and non-infectious diseases, thereby increasing veterinary costs (Rahman et al., 2022; Rahman et al., 2024). Buffaloes are susceptible to numerous diseases, in which bacterial and viral infections are major contributors to health losses, with individual diseases often showing morbidity exceeding 70% and mortality up to 25% (Samad, 2020; Ali et al., 2025). Limited awareness among farmers and insufficient diagnostic facilities hinder timely and proper treatment of diseases (Ahmed et al., 2025). Key viral, bacterial, and metabolic diseases that impair buffalo growth and reproductive performance are detailed in Tables 4, 5, and 6, along with their management strategies. Parasitic diseases also present a major challenge to buffalo production worldwide, including in Bangladesh (Biswas et al., 2021). The prevalence of parasitic infections is influenced by a combination of environmental factors such as ecological conditions, geographic location, and farm management practices, including housing, grazing, and deworming schedules. Other factors such as age, sex, body condition, parasite species, and parasite load also affect infection rates (Rizwan et al., 2024). The agroecological and geo-climatic characteristics of Bangladesh are highly conducive to the proliferation of parasites (Ali et al., 2020a; Biswas et al., 2021). The principal parasitic diseases affecting buffalo productivity are summarized in Table 7.

**Table 4:** Important viral diseases of buffalo.

Disease	Causative Agent	Transmission	External Factors	Clinical Signs	Morbidity and Mortality	Control & Prevention	References
Foot and Mouth Disease (FMD)	FMD virus (Picornaviridae)	Aerosol, ingestion, direct contact with infected animals/products	Temperature (20–34°C), humidity (50–60%), poor biosecurity	High fever, salivation, blisters on tongue, gums, teats, feet	Morbidity: ∼100%; Mortality: 20–25%	Vaccination, isolation, antimicrobials, and antiseptics (e.g., KMnO₄, glycerin)	Ali et al. (2020b); Paton et al. (2021)
Bovine Ephemeral Fever (BEF)	BEF virus (Rhabdoviridae)	Biting insects (mosquitoes), especially *Culex annulirostris*	Wet or dry seasons, hot and humid conditions, rainfall, and groundwater sources	Sudden fever (41°C), sharp milk drop, arthritis with a serofibrinous exudate, muscle swelling, mastitis	Morbidity: ∼100%; Mortality: 1–20%	Vaccination, vector control, NSAIDs, calcium borogluconate, antibiotics	Lee (2019); Martínez-Burnes et al. (2024)
Buffalopox	Buffalopox virus (Poxviridae)	Direct contact, biting insects as vectors (ticks, mosquitoes)	High humidity, poor hygiene, stress (transport, weather change)	Fever, pustular skin lesions on teats, udder, muzzle, ears, eyes	Morbidity: ∼70%; Mortality: 1–2%	Vaccination, strict biosecurity, vector control, topical antibiotics, NSAIDs	Lima et al. (2019); Kabir et al. (2023)
Bovine Viral Diarrhea (BVD)	BVD virus (Flaviviridae)	Vertical transmission, horizontal transmission via direct contact	Poor biosecurity, dirty pens and inadequate manure management	Mild diarrhea, reproductive loss, congenital defects (e.g., cerebellar hypoplasia), ataxia	Morbidity: 70–100%; Mortality: 1–50% (phase dependent)	Partial protection via vaccination; prevent carrier contact, supportive care	Primawidyawan et al. (2016); Martínez-Burnes et al. (2024)

**Table 5:** Important bacterial diseases of buffalo.

Disease	Causative Agent	Transmission	External Factors	Clinical Signs	Morbidity & Mortality	Control & Prevention	References
Haemorrhagic Septicemia (HS)	Pasteurella multocida (Gram-negative)	Ingestion/inhalation of contaminated feed, water, secretions or excretions	Stress, high humidity, and rainy weather	Fever, nasal discharge, hypersalivation, respiratory distress, Pharyngeal edema, sudden death	Morbidity: 60–80%, Mortality: 10–25%	Vaccination, early antibiotics (tetracycline, enrofloxacin), adjust based on AST	Moustafa et al. (2017); Abbas et al. (2018)
Anthrax	Bacillus anthracis (Gram-positive)	Ingestion of contaminated feed/water, entry via mucosal lesions	Climate shifts, alkaline calcium-rich soil, temp >15.5°C	High fever, hemorrhagic mucosa, dyspnea, bleeding from orifices, rapid death	Morbidity: 25–80%; Mortality: 2–20%	Vaccination, carcass disposal, disinfection, antibiotics with supportive care	Hassan et al. (2015); Alam et al. (2022)
Brucellosis	Brucella abortus (Gram-negative)	Contact/ingestion of infected fetal material, milk, aerosol, venereal	High-density herds, communal water sources	Abortions, retained placenta, infertility, mastitis in females; orchitis, epididymitis in males	Morbidity: 10–40%; Mortality: 2–5%	Vaccination, test and cull, biosecurity, tetracycline or streptomycin	Ghanbari et al. (2020); Khurana et al. (2021)
Mastitis	*Staphylococcus aureus, Streptococci, E. coli, Klebsiella, Bacillus spp.*	Contaminated milking equipment, direct contact	Poor housing, cylindrical teats, seasonal stress, dirty bedding	Fever, reduced milk, udder inflammation, altered milk, high SCC	Morbidity: 20–60%; Mortality: 1–3%	Hygiene, post-milking disinfection, antibiotics (ampicillin, sulfonamides), supportive care	Hoque et al. (2022); Chowdhury et al. (2024)

**Table 6:** Major metabolic disorders in buffalo.

Disease	Etiology	Predisposing Factors	Clinical Manifestations	Mortality	Control and Prevention	References
Milk Fever (Parturient Paresis)	Hypocalcemia at parturition.	High-calcium diets in prepartum period impair calcium mobilization	Anorexia, muscle tremors, incoordination, hypothermia (36.5–38°C), sternal or lateral recumbency, head turned toward the flank, flaccid paralysis	<10%	Prepartum low-calcium diet; oral calcium and vitamin D supplementation; intravenous calcium-magnesium therapy postpartum.	Kumari et al. (2022); Tufarelli et al. (2024)
Ketosis	Negative energy balance in early lactation, excessive fat mobilization	Energy-deficient or imbalanced diet, concurrent diseases, environmental or physiological stress	Reduced dry matter intake, decreased milk yield, ketone odor in breath, urine, and milk; elevated ketones; mucus-coated feces.	5–10%	Maintain energy-rich, balanced ration during transition; intravenous glucose administration; corticosteroid therapy (e.g., dexamethasone).	Bradford and Swartz (2020); Cascone et al. (2022)
Subacute Ruminal Acidosis (SARA)	Accumulation of volatile fatty acids due to excessive fermentable carbohydrates and inadequate fiber intake.	Rapid dietary transition to high-concentrate, low-forage diets, insufficient rumen buffering	Low appetite, decreased milk production, ruminal hypomotility, laminitis, soft feces with gas	<5%	Gradual adaptation to high-concentrate diets postpartum; adequate physically effective fiber; proper ration formulation.	Kitkas et al. (2019); Elmhadi et al. (2022)
Grass Tetany (Hypomagnesemia)	Hypomagnesemia due to low magnesium intake and absorption	Grazing on rapidly growing fertilized pastures, high lactational demand, stress	Hyperexcitability, muscle spasms, stiff gait, ataxia, tachypnea, convulsions, sudden death	Up to 40% if untreated	Oral or injectable magnesium supplementation; mineral-balanced rations; proper pasture management and stress reduction.	McCann et al. (2016); Tufarelli et al. (2024)

**Table 7:** Important parasitic diseases of buffalo.

Disease	Etiology	Transmission	External Factors	Clinical Signs	Mortality	Control and Prevention	References
Fasciolosis	*Fasciola gigantica, Fasciola hepatica*	Ingestion of metacercariae on plants or water via snail hosts (*Lymnaea, Galba*)	Presence of snail, marshy or waterlogged grazing areas, irregular deworming	Anemia, weight loss, submandibular edema, decreased milk, liver damage	<5%, increasing to 10–15% in severe cases	Routine deworming (Triclabendazole, Nitroxynil, Albendazole), drainage improvement, snail control	Hassan et al. (2020); Lalor et al. (2021)
Paramphistomosis	*Paramphistomum cervi, Paramphistomum microbothrium*	Ingestion of metacercariae from plants contaminated by cercaria from freshwater snails (*Planorbis, Lymnaea, Bulinus*)	High rainfall, dense snail populations, grazing behavior, irregular deworming	Anemia, hypoproteinemia, edema, diarrhea, anorexia, weight loss, rectal hemorrhage, decreased production	20–50% if untreated	Routine deworming (Oxyclozanide, Niclosamide), fluid/electrolyte therapy, avoid snail-infested grazing areas	Mohanta et al. (2017); Rafiq et al. (2020)
Ascariasis	*Toxocara vitulorum*	Transmammary, ingestion of infective eggs from contaminated feed, soil, or water	Poor sanitation, climatic factors, host age, and nutritional status	Swollen face, anorexia, colic, constipation, dehydration, weight loss, foul feces	35–50% in untreated calves	Routine deworming protocols (Ivermectin, Levamisole), good hygiene, and management	Biswas et al. (2021); Gholve et al. (2024)

## 8 Constraints and challenges in Buffalo production

Buffalo production in Bangladesh faces several constraints, including poor nutrition, reproductive inefficiency, low genetic merit, and health challenges, all of which adversely affect productivity and disease resistance. Dependence on low-quality natural pastures, particularly during the dry season, results in micronutrient deficiencies that reduce milk yield and prolong the calving interval (Habib et al., 2023). The absence of structured breeding programs, ineffective estrus detection, and limited use of artificial insemination (AI) further impair reproductive success by delaying puberty and reducing the rate of conception (Srirattana et al., 2022). Limited AI usage also restricts the introduction of superior germplasm from elite breeds such as Murrah and Nili-Ravi.

Another important issue is the management of disease. The prevalence of parasitic illnesses and endemic diseases like foot-and-mouth disease has increased due to inadequate veterinary infrastructure, low vaccination coverage, and a lack of awareness among farmers (Samad, 2020; Ali et al., 2020b). In addition, inadequate disease diagnosis and insufficient expert manpower negatively affect buffalo health (Ahmed et al., 2025). The severity of these problems is increased in remote and low-resource rural areas.

Genetic improvement is hindered by the lack of pedigree records, genomic tools, estimated breeding value (EBV) systems, and systematic performance recording (Rahman et al., 2020; Sarker et al., 2024). Reproductive challenges such as extended postpartum anestrus, subtle estrus expression, and seasonal breeding behavior further complicate breeding management (Jainudeen & Hafez, 2000; Perera, 2011). The lack of adequate infrastructure for AI and genomic selection continues to limit productivity gains in the absence of comprehensive national policies and integrated breeding programs (Bhuiyan et al., 2017).

### 8.1 Potential for improvement and future prospects

Despite these challenges, buffalo production in Bangladesh has great prospects for improvement and expansion. Ongoing government and non-governmental programs focusing on genetic enhancement, feed resource development, and farmer capacity building show promising results (El Debaky et al., 2019). Improved nutritional management, including the use of high-yielding fodder species, urea-molasses mineral blocks, and mineral supplementation, can substantially increase productivity (Hazra et al., 2014). High-yielding riverine breeds can be used in crossbreeding programs to improve feed conversion efficiency, growth performance, and milk yield (Islam et al., 2017; Karim et al., 2023). Sustainable genetic progress will require systematic genetic evaluation utilizing EBVs, genomic selection, and consistent performance recording (Sarker et al., 2024). Reproductive constraints such as seasonal breeding and poor estrus synchronization can be mitigated through hormonal protocols. Fixed-time artificial insemination (FTAI) protocols have demonstrated effectiveness in improving conception rates and reproductive efficiency (Table 8).

**Table 8:** Synchronization protocols and pregnancy rates in buffaloes.

Synchronization Protocol	Pregnancy Rate (%)	References
PGF2α	41	Jisna et al. (2024)
GnRH + PGF2α + GnRH	60	Gallab et al. (2022)
OVS + Resynch	84	Otava et al. (2021)
PGF2α	21	Atabay et al. (2020)
PGF2α + GnRH	37	Atabay et al. (2020)
PGF2α + GnRH	36	Atabay et al. (2024)
PGF2α + GnRH + HGG	39	Atabay et al. (2024)
CIDR + PGF2α	60	Amin et al. (2015)
CIDR + PGF2α	50	Samir et al. (2019)
CIDR + OVS	67	Samir et al. (2019)
PGF2α + GnRH + GnRH + PGF2α + GnRH	56	Waqas et al. (2016)
GnRH + PGF2α	100	Yendraliza et al. (2011)
CIDR	37	Naseer et al. (2011)
GnRH + PGF2α + GnRH	36	Warriach et al. (2008)

Protocols integrating Controlled Internal Drug Release (CIDR) devices with Gonadotropin-Releasing Hormone (GnRH)-based treatments consistently show improved pregnancy rates in buffaloes, including heat-stressed and anestrous animals. Such synchronization techniques can significantly enhance reproductive efficiency. With strategic implementation of these protocols and broader systemic reforms, Bangladesh’s buffalo industry has the potential to become a highly productive and sustainable sector, thereby supporting rural livelihoods and contributing to national economic development (Barile, 2005; Rahman et al., 2020).

### 8.2 Integration for sustainable buffalo development

A comprehensive strategy that connects productivity, reproduction, genetics, and health is necessary for the sustainable development of buffalo. According to De Rosa et al. (2009) and Borghese (2013), increasing animal health and well-being must coexist with improving genetic potential and reproductive efficiency to increase productivity. Selective breeding, improved nutrition, and disease prevention are all essential components of integrated management techniques. In addition, policy assistance is essential for giving farmers incentives, infrastructure, and training. According to Nanda & Nakao (2003), providing farmers with resources and information guarantees sustained growth and fortifies rural economies that rely on buffalo farming. A comprehensive strategy that integrates productivity, reproduction, genetics, and health is required for the sustainable development of buffalo. To increase resilience and lower costs in buffalo farming, recent methods have a strong emphasis on implementing sustainable practices such as rotational grazing, integrated pest management, and organic farming (EssFeed, 2024). The use of Integrated Farming Systems (IFS), which combine crops, livestock, aquaculture, and agroforestry, has been shown by Researchers to empower farmers and promote sustainable wealth. The International Buffalo Workshop 2024 in Bangladesh emphasized the significance of effective production, practical training for farmers and field veterinarians, and ways to mitigate climate change to create a sustainable buffalo milk value chain (UHB, 2024). Additionally, developing skills and guaranteeing the prosperity of buffalo farming communities depend on empowering buffalo farmers through focused training programs in animal health, reproductive technologies, and farm management (El Debaky et al., 2019; Jacobs, 2024a). Further enhancing the sustainability and effectiveness of buffalo agricultural operations are innovations like genetic breakthroughs and precision farming (Jacobs, 2024b; Chiariotti et al., 2025).

### 8.3 Nutritional and sanitary management of buffaloes in Bangladesh

Buffalo productivity in Bangladesh remains below potential, largely due to chronic nutritional deficiencies and poor hygienic conditions. Most smallholder farmers still depend heavily on low-quality roughages and crop residues, while seasonal shortages of green fodder further reduce nutrient availability. These feeding practices limit energy intake and restrict growth, milk yield, and reproductive efficiency (Chanda et al., 2021). Recently, field-level efforts have introduced more targeted feeding strategies. Precision supplementation using energy-protein concentrates and mineral mixtures formulated based on physiological requirements has led to visible improvements in body condition, metabolic status, and overall productivity (Faruque & Hossain, 2007; Mohd Azmi et al., 2021; Sultana et al., 2024). Alongside nutritional gains, improvements in sanitation and hygiene, including access to clean drinking water, improved waste drainage, and low-cost biosecure housing designs, have helped reduce disease burden and improve health outcomes (Jimenez et al., 2023).

However, the broader application of these strategies is limited by fragmented extension services, low farmer awareness, and poor adoption at the field level. To maximize the benefits of these interventions, a coordinated national approach linking evidence-based nutritional strategies with sanitary best practices and supported by farmer training and input access is essential to improve the sustainability and efficiency of buffalo production in Bangladesh.

### 8.4 Strategic interventions for improvement

To overcome the various challenges facing buffalo production in Bangladesh, it's essential to implement targeted, evidence-based strategies. These should be designed to address specific issues related to nutrition, reproduction, genetics, health, and policy support. Table 9 presents practical measures that can help improve productivity, boost reproductive performance, and strengthen herd health, which ultimately support the sustainable growth of the buffalo sector.

**Table 9:** Strategic interventions for improvement.

Area of Focus	Strategic Interventions	References
Nutrition	Distribution of urea-molasses mineral blocks; cultivation of high-yielding fodder (Napier, Berseem); use of balanced concentrate rations; routine mineral and vitamin supplementation.	Habib et al., 2020; Chaudhary et al., 2024
Reproduction	Adoption of estrus synchronization protocols (PGF2α, CIDR, GnRH-based); expansion of AI and FTAI programs; improved estrus detection methods; reproductive management training for farmers.	Choudhary et al., 2022; Hufana-Duran et al., 2025
Genetic Improvement	Structured crossbreeding using Murrah/Nili-Ravi semen; introduction of performance recording systems; use of genomic tools and estimated breeding values (EBV) for sire selection.	Rashid et al., 2019; Kumar et al., 2024
Health Management	Routine vaccination against endemic diseases (FMD, HS); targeted deworming protocols; mobile veterinary services; health education programs for smallholder farmers.	Samad, 2020; Ahmed et al., 2025
Infrastructure & Policy Support	Buffalo-specific extension services; farmer training in feeding, health, and reproductive management; support for buffalo milk marketing and value chain development; national buffalo breeding policy.	El Debaky et al., 2019; Jacobs, 2024a

## 9 Conclusions

The buffalo sector in Bangladesh holds considerable potential to boost milk and meat production through targeted genetic improvement and crossbreeding with high-yielding riverine breeds such as Murrah and Nili-Ravi. These interventions have shown promising results in enhancing milk yield and growth rates. However, the lack of structured breeding programs, systematic genetic evaluation, performance recording, and reproductive biotechnologies continues to hinder sustainable progress. To fully harness the genetic potential of the national buffalo herd, it is essential to implement comprehensive breeding strategies that incorporate tools such as estimated breeding values and genomic selection. Equally important is the integration of strong animal health management through regular vaccinations, health monitoring, biosecurity measures, and parasite control to reduce mortality, improve feed efficiency, and extend the productive lifespan of buffalo. This review provides strategic insights for stakeholders, policymakers, researchers, extension agents, and farmers, both nationally and globally, offering a foundation for evidence-based interventions and policies aimed at building a productive, resilient, and health-optimized buffalo sector in Bangladesh. Collaborative efforts among government agencies, research institutions, NGOs, and farming communities will be crucial for achieving sustainable genetic and productivity gains.

## 10 Recommendations

To achieve sustainable buffalo production in Bangladesh, the following measures are recommended:

I. Develop and enforce a national buffalo breeding strategy incorporating systematic performance recording and genomic selection methodologies.
II. Expand artificial insemination and estrus synchronization programs through effective public-private partnerships and comprehensive farmer capacity-building.
III. Strengthen animal health and extension services to improve disease prevention, reproductive efficiency, and herd productivity.
IV. Promote climate-smart, community-based farming systems to enhance production efficiency and environmental resilience.
V. Facilitate collaboration among government bodies, non-governmental organizations, research institutions, and farmers to ensure broad adoption of advanced reproductive technologies and best management practices.

## Funding statement

No funding was received for this research.

## Consent for publication

Not applicable.

## Declaration of generative AI and AI-assisted technologies in the writing process

During the preparation of this manuscript, the authors used ChatGPT (free version) to improve language clarity and readability. All use was conducted under human oversight, and the outputs were critically reviewed and edited by the authors to ensure the accuracy and integrity of the scientific content

## Ethical statement

This review article is based solely on previously published data and does not involve any original research involving animals or human subjects. Therefore, ethical approval was not necessary.

## CRediT authorship contribution statement

**Eshtiak Ahamed Pehan:** Writing – review & editing, Writing – original draft, Visualization, Methodology, Conceptualization. **Manik Miah:** Writing – review & editing, Writing – original draft, Visualization, Resources. **Md Habibur Rahman:** Writing – review & editing, Writing – original draft, Visualization, Supervision, Methodology, Conceptualization. **Shahanaj Ferdousi Shejuty:** Writing – original draft, Visualization, Resources. **Md Nurul Haque:** Writing – original draft, Resources. **Md Nazmul Huda:** Writing – original draft, Resources. **Md Rezwanul Habib:** Writing – review & editing, Writing – original draft, Supervision. **Md Younus Ali:** Writing – review & editing, Writing – original draft, Visualization, Supervision, Conceptualization.

## Declaration of competing interest

The authors declare that they have no known competing financial interests or personal relationships that could have appeared to influence the work reported in this paper.

## Data Availability

Data sharing is not applicable to this article.
